# Effectiveness of a specific physical therapy program for Charcot-Marie-Tooth on sleep quality, pain perception, and nocturnal cramps: a pilot study

**DOI:** 10.5935/1984-0063.20220058

**Published:** 2022

**Authors:** Cynthia Coelho Souza, Julia Ribeiro da Silva Vallim, Eduardo Luis de Aquino Neves, Paula Santos Nunes, Iandra Maria Pinheiro de França Costa, Lidiane Carine Lima Santos Barreto, Catarina Andrade Garcez, Adriano Antunes de Souza Araujo

**Affiliations:** 1 Universidade Federal de Sergipe, Departament of Pharmacy - Aracaju - Sergipe - Brazil; 2 Universidade Federal de São Paulo, Departament of Psychobiology - São Paulo - São Paulo - Brazil

**Keywords:** Musculoskeletal and Neural Physiological Phenomena, Actigraphy, Hereditary Sensory and Motor Neuropathy, Rehabilitation, Physical Therapy Modalities

## Abstract

**Introduction:**

Chronic pain, nocturnal cramps, and sleep alterations are prevalent symptoms and signals in Charcot-Marie-Tooth disease patients. Sleep and pain are bidirectionally related and physical therapy can improve the binomial sleep and pain/nocturnal cramps. Therefore, we hypothesized that the application of a specific physical therapy program for Charcot-Marie-Tooth disease would improve sleep quality, pain perception, and nocturnal cramps.

**Material and Methods:**

A non-randomized controlled study that included 9 Charcot-Marie-Tooth disease patients (intervention group - physical therapy program) and 8 controls (active control group - booklet on sleep hygiene). The intervention lasted 8 weeks, three sessions per week. The effects were evaluated ten days before (baseline) and ten days after the intervention (post). Our primary outcome was sleep quality (subjective and objective, assessed by Pittsburgh sleep quality index and actigraphy, respectively); and secondary outcomes were pain perception (brief pain inventory) and nocturnal cramps (self-report).

**Results:**

The program was able to improve the subjective sleep quality (*p*=0.005) and nocturnal cramps (*p*<0.001) but had no effect on actigraphy data (*p*>0.05) neither on pain perception (*p*>0.05).

**Conclusion:**

Our initial hypothesis was partially corroborated: the improvement in subjective quality of sleep and nocturnal cramps is already beneficial for the health promotion of the volunteers in this study affected by the disease. Our findings may serve as a basis for future research to develop a program focused on the treatment of analgesia, which can improve pain perception and alter the objective quality of sleep.

## INTRODUCTION

Charcot-Marie-Tooth disease (CMT) is a hereditary peripheral neuropathy, characterized by sensory and motor manifestations^1^,^2^ and was first described by Jean-Martin Charcot, Pierre Marie, and Howard Henry Tooth in the 19^th^ century^3^,^4^. It is a progressive disease that initially affects the lower limbs with symptoms that include feet deformity and leg atrophy^1^,^5^.

Another symptom of the CMT disease is the neuropathic pain that affects 23 to 100% of the individuals with the disease^6^. It affects mainly the extremities of the body (feet and hands) and can also manifest itself as muscle cramps^6^,^7^, which in CMT disease occurs mainly at night^8^. These nocturnal symptoms compromise their quality of life and interfere in daily activities such as sleep and ability to perform exercises^8^.

The study by Ribiere et al. (2012)^9^ demonstrated that about 63% of the pain cases in CTM disease are neuromuscular related, with an average duration higher than 15 years, which indicates a chronic pain condition.

Chronic pain and sleep are bidirectionally related: proper sleep improves physical and psychological symptoms of pain and pain worsens sleep quality, but the mechanisms involved in this relationship are not well established^10^,^11^. In a previous study conducted by our group, we observed that pain perception, fatigue, and nocturnal cramps were related to sleep alterations in patients with CMT disease^12^ and that these patients present greater sleep fragmentation and changes in its architecture^13^.

Given this evidence, we hypothesized that the application of an intervention capable of improving pain symptoms could improve sleep and, consequently, improve the patients’ quality of life. Physical therapy is indicated and used to treat neuropathies as it can promote functionality gain^14^ and is also used in sleep medicine as a complementary treatment for several respiratory and neurological-based disorders^15^.

Therefore, physical therapy could contribute positively to the sleep-pain binomial in patients with CMT disease, allowing them to recover their physical functionality and improving their ability to perform daily tasks.

The objective of this study was to evaluate the effects of a specific physical therapy program directed to CMT disease (SPTP-CMT) on sleep, pain perception, and nocturnal cramps in patients with Charcot-Marie-Tooth disease. We hypothesize that physical therapy will be able to improve sleep quality (subjective and objective), pain perception, and nocturnal cramps in these patients.

## MATERIAL AND METHODS

A non-randomized controlled trial conducted between October and December 2017 in three cities of Sergipe - Brazil: Tobias Barreto, Pedrinhas, and Cristinápolis. Volunteers were recruited in partnership with the public health system and the study only began after approval by the ethics committee according to Resolution No. 466/12 (No. CAAE: 69490117.5.0000.5546). This study was registered in the Brazilian Registry of Clinical Trials (RBR-6hyt5g7) and its design and conduct following the methods described by CONSORT.

**Inclusion criteria:** individuals aged between 16 and 65 years, with a clinical and electrophysiological phenotype consistent with the disease. The diagnosis was made by clinical and electroneuromyographic examination, in addition to the evaluation of the CMTNS score^16^. The control group was composed of individuals from the same family not diagnosed with the disease, matched for sex and age.

**Exclusion criteria:** acute or chronic lung disease, cognitive or psychiatric disorders, severe or poorly controlled hypertension, heart failure, chronic kidney disease, systemic diseases, diabetes mellitus, pregnant women, obesity, active smokers, alcoholics, and refusal to participate in the study.

**Sample size calculation:** we performed a sample size calculation using the *GPower 3.1.9.7* software, considering an effect size of 33%, power of 80%, and alpha of 5%, with two timepoints and two evaluation groups, obtaining a minimum value of 22 individuals (11 per group).

### General procedures

An initial assessment was performed to collect demographic data such as name, gender, date of birth, age, address, investigation regarding the use of medications, associated diseases, and anthropometry. A targeted CMT disease score was recorded to assess disease severity as described by Shy et al. (2005)^16^: results below 10 were considered as mild, between 11 and 20 points as moderate and above 21 as severe.

**Primary outcomes:** subjective and objective sleep quality.

**Secondary outcomes:** pain perception and nocturnal cramps.

#### Subjective assessments

Nocturnal cramps: was based on self-report, the volunteers received an evaluation form in which they had to rate the intensity of the cramps from 0 to 10.

Pittsburgh sleep quality index (PSQI): subjective assessment of sleep quality^15^. Results less than or equal to 5 were considered good sleep quality and greater than 5 poor sleep quality. Was applied the version validated for Portuguese-BR^17^,^18^.

Epworth sleepiness scale (ESS): evaluates the chance of sleeping in everyday situations^17^,^19^. Scores less than 10 were considered as normal daytime sleepiness, and greater than or equal to 10 as excessive daytime sleepiness. Was applied the version validated for Portuguese-BR^19^,^20^.

Brief pain inventory (BPI): assesses the perception of pain^19^. Results equal to 0 were considered as no pain; between 1 and 4 points as mild pain; between 5 and 6 points as moderate pain and between 7 and 10 points as severe pain. Was applied the version validated for Portuguese-BR^21^,^22^.

Chalder fatigue scale (CFS): assesses the perception of fatigue. Results lower than 4 were considered as absent fatigue and higher or equal to 4, as present fatigue. Was applied the version validated for Portuguese-BR^23^,^24^.

#### Objective assessments

The actigraph model used in this study was the Mini Motionlogger Actigraph - Basic 32 (Ambulatory Monitoring Inc., USA) with the ZCM algorithm (“zero crossing mode”). The time of collection was set at 1 minute. The computerized analysis was done according to the algorithms proposed by Cole et al. (1992)^25^ and Sadeh et al. (1994)^26^, available in the programs Action 3 - Version 3.15 and Action for Windows - Version 1.05 (AMA, USA). The estimated parameters were sleep latency, sleep efficiency, total sleep time, and wake time after sleep onset (WASO).

Actigraphy data analysis was carried out by an individual with expertise in the field for at least ten years and reviewed by the second author of this paper, who is also an expert in the analysis of this type of data. During the recording period, the volunteers also filled out a sleep diary used for combined actigraphy analysis.

### Specific physical therapy program for Charcot-Marie-Tooth

Individuals participated in a specific physical therapy program for CMT disease (SPTP-CMT). The program was divided into two phases and consisted of exercises targeting four domains: A) gain of joint range of motion in lower limbs; B) muscle strengthening in proximal and mid-limb segments; C) coordination, static and dynamic balance; and D) functional independence. The intervention lasted 8 weeks, totalizing 24 sessions, which were held three times a week, each session lasting an hour.

Before the physical therapy sessions began, the volunteers performed an initial warm-up series: going up and down a ramp five times and pedaling on an exercise bike for five minutes. Then, in a sitting position, they received pressure on the soles of the feet with a six-centimeter diameter proprioception ball to release the plantar fascia.

The 24 sessions were divided into two exercise phases: phase 1 up to the tenth session) and phase 2 (remaining fourteen sessions).

#### Phase 1

**A) Stretching** (performed in three sets of 15 seconds for each muscle group).

Hamstring stretching performed with the volunteer in dorsal decubitus position with a stretching band supported on the midfoot for hip flexion with knee extension. The contra lateral limb remained supported in flexion on the stretcher.

Stretching of eversors, inversors, plantar flexors, and dorsiflexors: sitting on a chair, the volunteers performed the self-stretching with the stretching band of eversors, inversors, plantar flexors, and dorsiflexors.

Triceps sural stretching: the volunteer stood in a standing position with the hip and knee in extension and the foot resting on the floor, the opposite side with knee flexion and the hands resting on the wall.

Iliopsoas stretching: the last stretching series performed was the standing quadriceps and iliopsoas stretching, with knee flexion, gluteal contraction, and support for execution.

**B) Muscle strengthening** (performed in three sets of 10 repetitions).

Gluteal strengthening: the volunteer was in dorsal decubitus, performing hip elevation with bent knees and feet supported on a stable surface (bridge).

Strengthening of dorsiflexors and eversors: the volunteers were in a sitting position and performed the exercise from the movement against elastic band resistance.

Strengthening the quadriceps: in the sitting position, the volunteers performed a knee extension with a shin pad.

Ischiostibial strengthening with the volunteer in standing position, knee flexion was performed, with shin pads.

**C) Coordination, static and dynamic balance**

Proprioception exercises on a proprioceptive board, standing up, performing plantar flexion, and dorsiflexion for two minutes. Next, the unipodal support on a stable surface with and without visual feedback and, finally, the exercise of sitting and standing on a Swiss ball/chair, with or without support.

#### Phase 2

**A) Stretching** (performed in 15 seconds standing + 15 seconds with torso flexion).

Triceps sural and hamstring stretching: the volunteer was positioned standing on an incline.

Quadriceps and iliopsoas stretching: the volunteer stood with one of the lower limbs on the stretcher, with the knee flexed, and the contralateral limb with the foot on the floor and the knee flexed.

**B) Muscle strengthening** (performed in three sets of 10 repetitions).

Strengthening the quadriceps, gluteal muscles, and posterior thigh: with the individual lying in a supine position in dorsal decubitus with knees bent and support of the plantar part of the feet on the stretcher raising the glutes on the stretcher.

**C) Coordination, static and dynamic balance**

1. Plantar flexion and dorsiflexion for two minutes on a proprioceptive standing board.
2. Squatting with support of only one upper limb (if you could perform without support) on foam with a density of 60 cm,
3. Stepwise advance on a proprioceptive disk.

### Experimental design

Baseline (ten days before the beginning of the intervention)

On day 0 (D0) the volunteers filled out the questionnaires, started the actigraphy, received the sleep diary. After ten days, the actigraphs were removed, sleep diaries collected, and the intervention started (D10). The control group also received a lecture on sleep hygiene and printed material on the subject. The research team contacted the members of this group weekly, via telephone, to monitor their sleep habits.

Post-intervention (10 days after the end of the intervention)

Ten days after the end of the intervention, the volunteers started again the actigraphy recording, filled the questionnaires and the sleep diary.

No follow-up was performed in this study.

### Statistical analysis

The general mixed model (GMM) was applied, considering as dependent variables the scores in the questionnaires and the parameters obtained by the actigraphy recording. The group (control/physical therapy; reference - control), gender (female/male; reference - male), time (baseline and post-intervention; reference - baseline), and the interaction between group and time were considered as fixed factors, and age and BMI as covariates in the model.

Individuals were added as a random factor to verify the effect of intra-individual variability (placebo effect). This choice was made based on theory (the effects would not come from the intervention per se, but from the manipulation, hosting of the research team, etc.) and on the analysis of components of variance (ICC>10%)^27^.

The post hoc test adopted was Bonferroni and the significance level was 5% (*p*<0.05). The software used for the analyses was Jamovi version 1.2.27. Effect size was calculated using Lenhard e Lenhard (2016)^28^ calculator.

Treatment effect: the treatment effect was calculated using the formulas described by Herbert (2000)^29^. Since our study was designed as a non-randomized trial, we considered as reference values of the pre-interventions assessments. The values were calculated individually according to the following formula: (a_pre_ - a_post_)/a_pre_; and, to describe the percentages of the treatment effect, these values were multiplied by 100. Negative values indicate higher values after the intervention and positive ones, the opposite.

## RESULTS

**Figure 1:**
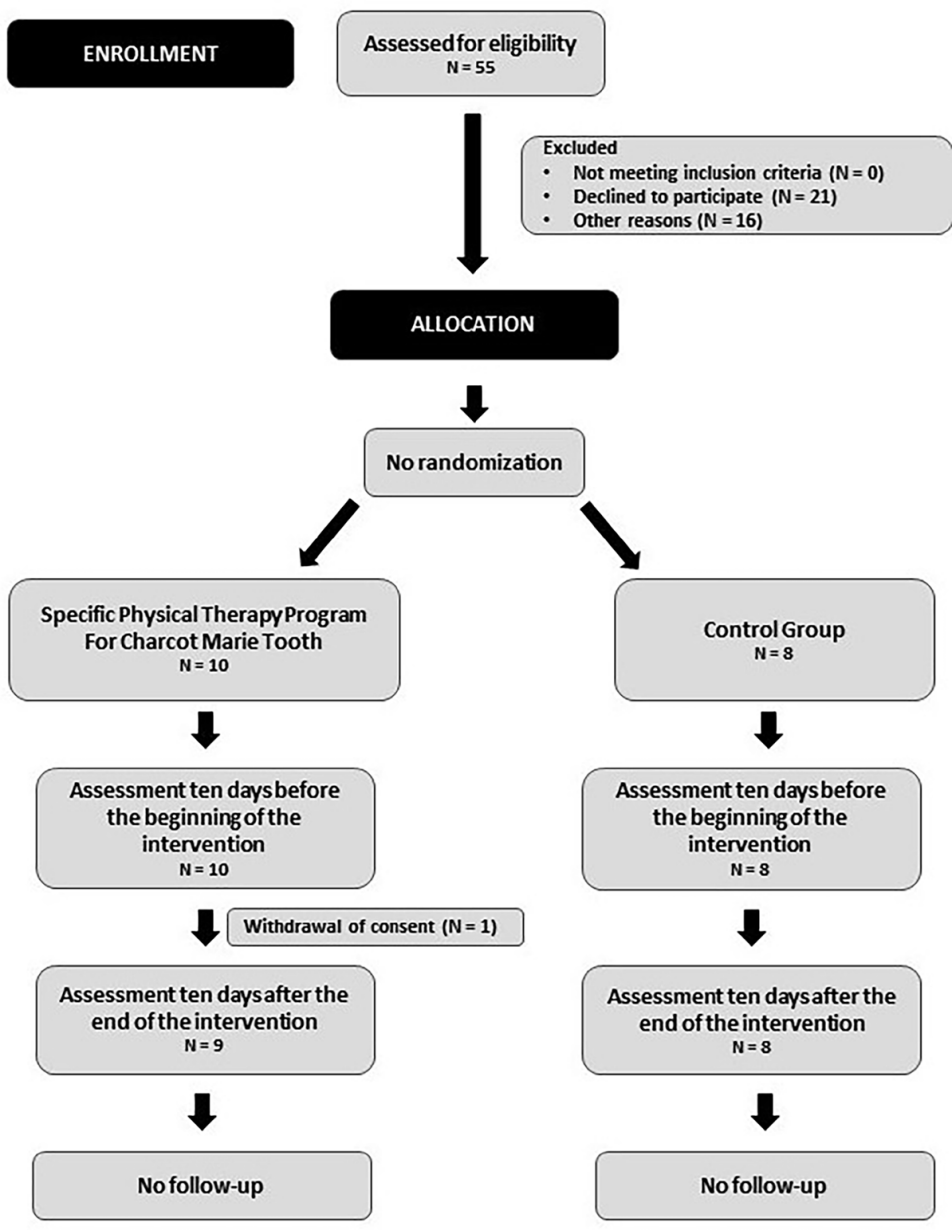
Flow chart of the procedures performed in this study.

Table 1 shows the sociodemographic data of our sample and Figure 1 the flow chart of the procedures performed in the study. During the intervention, there was the loss of only one female volunteer who withdrew her consent, so her data was excluded from the analyses.

**Table 1:** Sociodemographic data of the sample. Numerical data are represented as mean ± standard deviation and categorical data as absolute frequency.

Variables	CT N=8	SPTP-CMT N=9
**Age (years)**	33.9 ± 7.6 (range 26 - 46)	42.3 ± 12.9 (range 23 - 56)
**BMI (kg/m^2^)**	23.0 ± 1.7	27.2 ± 2.9
**Gender**	3 women 5 men	7 women 2 men
**Disease**	Without disease	CMT type 1
**Disease severity (CMTNS)**	Non-existent	2 mild 7 moderate

### Primary outcome - sleep quality

**Figure 2:**
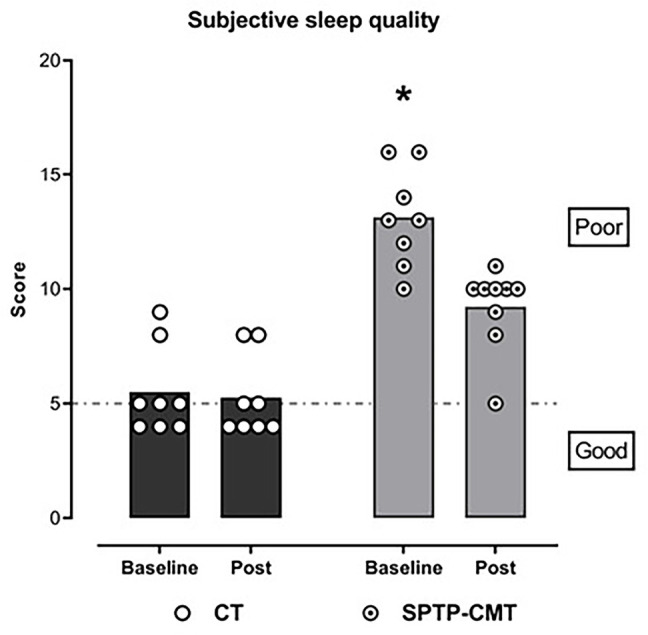
Effect of the specific physical therapy program for CMT disease on subjective sleep quality. Each symbol represents the individual scores, the bars the means, the dotted line the cutoff point for the categories on the right.

Notes: CT = Control group; SPTP-CMT = Intervention group; *General Mixed Model (GMM) (*p*<0.05). Higher score than the other groups and measures.

Physical therapy was able to improve subjective sleep quality (*p*=0.005; β=3.4 95%CI: 1.0-1.4; ICC=51, *d*=2.0 - large effect 95%CI: 0.4-3.6) when compared to the control group (Figure 2).

To evaluate the daytime consequences of the program on sleep, we also applied the Epworth sleepiness scale. We did not observe a significant effect of physical therapy on daytime sleepiness (*p*=0.99, ICC=54%) (Table 2).

**Table 2:** Results of daytime sleepiness, pain perception and fatigue questionnaires. Results are presented as mean ± standard deviation.

Variables	CT		SPTP-CMT	
	Baseline	Post	Baseline	Post
**Daytime sleepiness (ESS)**	11.4 ± 4.6	9.6 ± 2.9	9.0 ± 4.5	7.2 ± 2.8
**Brief pain inventory (BPI)**	2.9 ± 1.7	2.8 ± 1.8	4.2 ± 1.6	3.3 ± 1.1
**Chalder fatigue scale (CD)**	1.5 ± 1.2	0.5 ± 0.8	3.7 ± 2.9^a^	2.9 ± 2.2^a^

Notes:

a General Mixed Model (GMM) (*p*<0.05). Higher score than the control group.

We did not observe a significant effect of the intervention on the sleep parameters obtained from actigraphy: latency (*p*=0.22 ICC=2.7.10^-15^%), efficiency (*p*=0.94; ICC=34%), total sleep time (*p*=0.84, ICC=0%), and awake time after sleep onset (*p*=0.59, ICC=26%) (Table 3).

**Table 3:** Sleep parameters obtained from actigraphy. Results are presented as mean ± standard deviation.

Variables	CT		SPTP-CMT	
	Baseline	Post	Baseline	Post
**Latency (minutes)**	12.0 ± 6.5	9.3 ± 3.4	9.3 ± 7.8	14.0 ± 11.4
**Efficiency (%)**	91.5 ± 3.7	92.1 ± 4.0	93.6 ± 3.9	93.7 ± 3.1
**Duration (hours)**	5.0 ± 1.2	6.0 ± 2.2	5.8 ± 1.2	6.4 ± 2.6
**WASO (minutes)**	30.2 ± 17.9	23.8 ± 14.8	26.1 ± 12.5	24.9 ± 15.0

### Secondary outcomes - pain perception and nocturnal cramps

**Figure 3:**
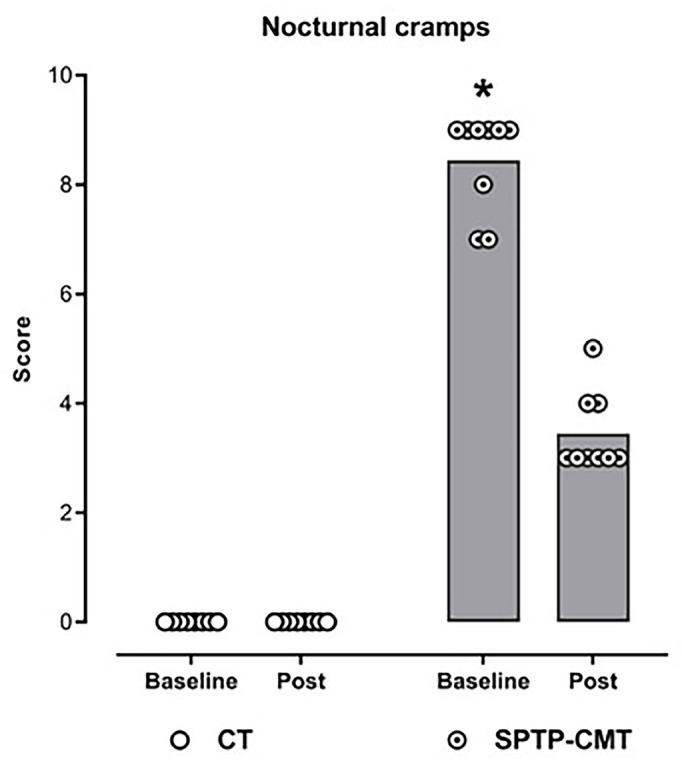
Effect of the specific physical therapy program for CMT disease on nocturnal cramps. Each symbol represents the individual scores, the bars the means, the dotted line the cutoff point for the categories on the right.

Notes: CT = Control group; SPTP-CMT = Intervention group; *General Mixed Model (GMM) (p<0.05). Higher score than the other groups and measures.

Physical therapy was able to improve nocturnal cramps when compared to the control group (*p*<0.001, β=5.0, 95%CI: 4.4-5.6, ICC=44%, *d*=6,2 - large effect 95%CI: 3.0-9.3) (Figure 3).

The program had no effect on pain perception (*p*=0.36, ICC=45%) and on fatigue (*p*=0.84, ICC=13%). For fatigue, a significant effect of the group factor alone was observed (*p*<0.001, β=-4.3, 95%CI: -6.3 - -2.4, ICC=13%): regardless of the intervention patients diagnosed with CMT disease had worse fatigue perception than the control group (Table 2).

### Treatment effects

**Figure 4:**
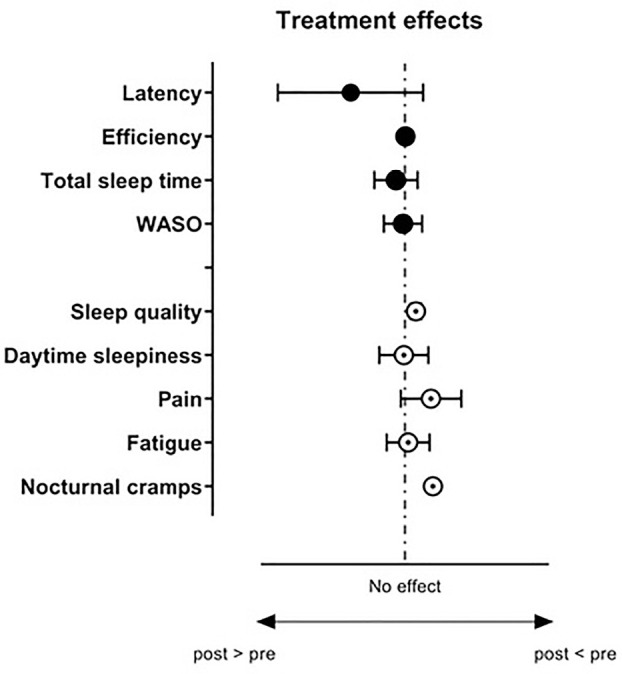
Forest plot of the treatment effects on the outcomes. Each symbol represents the mean value and the error bars the 95% confidence interval.

As can be seen in Figure 4, the specific physical therapy program showed a significant effect only for the subjective assessments of nocturnal cramps and subjective sleep quality, with effects of about 59% and 23%, respectively (Figure 4).

The minimal clinically important difference for chronic pain varies considerably and there is no current agreement on a threshold value. Olsen et al. (2018)^30^ suggest adopting a mean value of 32%. Despite large intra-individual variability, we observed an average reduction of about 55% in pain (Figure 4), which can be considered a minimal change to be clinically relevant.

## DISCUSSION

The results obtained in this study partially corroborated the proposed hypothesis. The specific physical therapy program improved the subjective sleep quality and nocturnal cramps (Figures 2 and 3) but did not show a significant effect on the objective sleep parameters and pain perception (Tables 2 and 3).

Eadie et al. (2013)^31^ obtained similar results: improvement in the subjective sleep quality after three and six months of intervention in people with chronic low back pain with a similar intervention protocol and ways of assessing the outcomes (Pittsburgh sleep quality index and actigraphy) but with some differences mainly in the duration of the intervention (8 weekly meetings *vs.* 3 weekly meetings performed in our study).

It has also been observed that stretching before bed has been able to improve nighttime cramps by reducing their frequency and intensity. In our study we did not assess frequency, but we obtained similar results, with a reduction in the intensity of nighttime cramps in patients with CMT disease, but it is worth noting important differences in the application of the intervention (time and duration of the intervention)^32^.

To our knowledge, this is the first study whose objective was to evaluate the effect of physical therapy on sleep in patients with CMT, so evidence for comparison with our outcome is lacking. Studies indicate that physical therapy is considered a useful tool for symptom management in CMT disease since it can improve the patient’s muscle functionality of patients^33^. However, it is worth noting that there are a wide variety of outcomes and intervention protocols, which hinders a more effective match with our results^33^.

Despite this subjective improvement in sleep quality, the same was not observed in the objective measurements obtained from the actigraphy recording (Table 3 and Figure 4). Wang and Boros (2019)^34^ demonstrated that moderate physical exercises are more effective in improving sleep quality than intense ones.

The program also had no effect on daytime sleepiness (Table 2 and Figure 4). Although exercise can alter sleep architecture and improve daytime sleepiness^15^,^31^,^34^, we did not observe this effect. We attribute this result to the lifestyle of the volunteers, who are from cities in the interior of Sergipe, and were able to take daytime naps, which may have impacted their sleepiness.

The ICC values indicate that our results also showed high intra-individual variability, which may be an indication of a placebo effect. Studies show that for analgesia this response may be caused by the expectation of change in symptoms combined with emotional motivations^35^. This may explain the absence of improvement in actigraphy parameters.

Sleep is regulated by biological factors (sleep pressure and circadian rhythm) but also by social and environmental factors, which influence sleep duration. Interestingly, the sleep patterns of the volunteers in our study are like ancestral sleep theories: sleep during the night of 5 to 7 hours and naps distributed throughout the day^36^,^37^.

Data in the literature show that rehabilitation programs that use physical therapy as a foundation (e.g., stretching exercises and body awareness) are the most effective for the mid-to-long-term treatment of fatigue^38^. In this work, we found no significant effect of the program on perceived pain and fatigue. One possible factor is that perhaps the duration of the intervention was not enough to lead to these modifications since a study by Eadie et al. (2013)^31^ observed effects after three and six months of intervention.

It is also worth noting that although the physical therapy program used in this study was based on the joint strengthening of the lower limbs, there was no region-specific stimulation or focus on systemic condition. Studies show that depending on the type of peripheral pain, stimulation can be done on the skin with different materials to reduce the sensitization of peripheral nerves^39^,^40^.

Therefore, further studies evaluating the effects of physical therapy, adopting different protocols (e.g., increasing the intervention time, tracking the evolution of symptoms over weeks, or focusing on analgesia) may answer this gap in the literature and offer more conclusive data.

### Study limitations

A valid limitation to be pointed out is the sample size. We know the importance of adequate sample size, but due to the characteristics of the disease (uncommon) and the difficulty in adhering to the physical therapy program, we were not able to fit the sample to the ideal size. Since this is a pilot and preliminary study, future studies with a more adequate sample size will be able to better elucidate the relationship between sleep and pain, and the possible effects of physical therapy on these parameters.

Other limitation is the lack of randomization since a non-randomized allocation can lead to biased interpretation of the data. This does not invalidate our results, as non-randomized studies are considered moderate level of evidence^41^. We tried to reduce these imbalances by using an age- and sex-matched control group and controlling for some variables in the statistical analysis^42^ but we are aware of this limitation.

## CONCLUSION

Our hypothesis was partially corroborated by our results: the specific physical therapy program for Charcot-Marie-Tooth was able to improve subjective sleep quality and nocturnal cramps, but we did not observe an effect on objective sleep parameters, assessed by actigraphy.
